# The advantage of self-protecting interventions in mitigating epidemic circulation at the community level

**DOI:** 10.1038/s41598-022-20152-4

**Published:** 2022-09-24

**Authors:** Romualdo Pastor-Satorras, Claudio Castellano

**Affiliations:** 1 Departament de Física, Universitat Politècnica de Catalunya, Campus Nord B4, 08034 Barcelona, Spain; 2 Istituto dei Sistemi Complessi (ISC-CNR), Via dei Taurini 19, I-00185 Rome, Italy; 3 Centro Ricerche Enrico Fermi, Piazza del Viminale, 1, I-00184 Rome, Italy

**Keywords:** Nonlinear phenomena, Statistical physics, Biological physics

## Abstract

Protecting interventions of many types (both pharmaceutical and non-pharmaceutical) can be deployed against the spreading of a communicable disease, as the worldwide COVID-19 pandemic has dramatically shown. Here we investigate in detail the effects at the population level of interventions that provide an asymmetric protection between the people involved in a single interaction. Masks of different filtration types, either protecting mainly the wearer or the contacts of the wearer, are a prominent example of these interventions. By means of analytical calculations and extensive simulations of simple epidemic models on networks, we show that interventions protecting more efficiently the adopter (e.g the mask wearer) are more effective than interventions protecting primarily the contacts of the adopter in reducing the prevalence of the disease and the number of concurrently infected individuals (“flattening the curve”). This observation is backed up by the study of a more realistic epidemic model on an empirical network representing the patterns of contacts in the city of Portland. Our results point out that promoting wearer-protecting face masks and other self-protecting interventions, though deemed selfish and inefficient, can actually be a better strategy to efficiently curtail pandemic spreading.

## Introduction

The recent COVID-19 pandemic has shown how fragile world societies are when confronted to the runaway spreading of a new virus strain^1^. In stark contrast with other diseases, however, the combination of recent scientific advances, liberal funding by governments and the availability of large scale manufacturing, has allowed to create and deploy world-wide vaccines for the SARS-CoV-2 virus at unprecedented speed^2^, a fact that has without any doubts greatly diminished the death toll expected in this pandemic. Despite this huge effort, however, more than nine months lapsed between the declaration of the COVID-19 pandemic by WHO in March 2020 and the approval of the first vaccine in December 2020. The large-scale deployment of vaccines worldwide required additional months and is still an unsolved problem in many areas of the planet. Moreover, the protection guaranteed by vaccines against recently emerged new variants is far from perfect. In this context, societies have had and still have to fight the propagation of the virus by adopting also more traditional non-pharmaceutical interventions^3^. These interventions, aiming at limiting the spread of a disease^1^, include collective actions, such as school closures, prohibition of large meetings, closing of public transportation, curfews or lockdown orders^4^. At an individual level, non-pharmaceutical interventions include, among others, physical distancing, hand and respiratory hygiene, and face masks. Understanding the impact of each of these interventions at the level of the single transmission event among individuals and how this translates at the community level is a crucial goal, which has attracted a huge scientific interest^5–17^.

In this paper we present an analysis of how the reduction of the chance that the disease is transmitted in a single contact, induced by a generic type of protecting intervention, results in an overall mitigation of the epidemic circulation in the population. In particular, we focus on asymmetric interventions having different efficacies at the individual level, i.e., in reducing the infectivity of an infected individual with respect to decreasing the susceptibility of a susceptible one.

As one of the most widely adopted means to fight the current pandemic, mask wearing is the paradigmatic example of these types of interventions. Given the evidence for the airborne transmission of SARS-CoV-2 by means of droplets and aerosols^18^, recent research has shown that face masks are a very effective way to reduce the spread of COVID-19^19–26^. Different types of masks exist, characterized by very different protecting performance, both qualitatively and quantitatively^27,28^. Some protect mainly the mask wearer, such as valved masks endowed with an exhalation valve. Others mainly protect the contacts of the mask wearer, such as surgical or cloth masks. Such asymmetric efficacy is however more general. For example, vaccines reduce the susceptibility of individuals to being infected upon contact with an infectious person, but they need not be equally effective in reducing the capability of vaccinated infected individuals to transmit the pathogen further if they ever catch the disease^29^. Also, symptomatic treatment reducing cough and sneezing hinders the possibility to transmit the disease onward but it does not reduce the chance to be infected.

Here we present a comparative analysis of the global efficiency of generic protective interventions (PI) in the context of epidemic models on networks^30^, representing the patterns of social contacts among people that mediate the propagation of an infective pathogen^31^. We use a formulation over networks with a static topology, parametrizing the effect of PIs in terms of the fraction of adopters and of the efficacy of adoption in reducing the chance to receiving/transmitting the disease.

We analyze the effect of different types of PIs at the community level by considering two paradigmatic models of disease propagation in networks^32,33^: the Susceptible-Infected-Removed (SIR) model for non-recurrent diseases, that confer immunity and develop in outbreaks (similarly to COVID-19, at least on short time scales), and the Susceptible-Infected-Susceptible (SIS) model for recurrent diseases that may lead to a steady endemic state. Using a combination of numerical simulations and analytical calculations we show that the adoption of asymmetric PIs leads to surprisingly different scenarios. In particular interventions protecting the adopter turn out to have a stronger effect at the population level. If PIs of the same efficacy in reducing infectivity/susceptibility are available, it is better to use PIs protecting the adopter, as they suppress more the global circulation of the disease. We investigate the origin of this unexpected finding and show that the conclusion holds also when considering more realistic interaction patterns and disease dynamics.

## Results

### Modeling the effect of protecting interventions

The effect of protecting interventions on the propagation of a disease can be modeled by considering the modification of the probability that the disease is transmitted between an infected and a susceptible individual^20,34^. Consider a population of *N* individuals, whose pattern of contacts is determined by a complex network^35^, in which nodes represent individuals and edges the presence of social contacts between pairs of individuals. The network is fully described by its adjacency matrix $a_{ij}$, taking value 1 if nodes *i* an *j* are connected, and zero otherwise. From a statistical perspective, the network can be characterized by its degree distribution *P*(*k*), representing the probability that a randomly chosen node has degree *k* (i.e., it is connected to *k* other individuals).

Disease transmission is mediated by contacts between infected and susceptible individuals. The properties of this transmission are mathematically encoded^32,33^ in terms of an infection rate $\beta$, representing the probability per unit time that the infection is transmitted from an infected to a susceptible along a single contact. Protecting interventions induce a reduction of this infection rate in two possible manners. An intervention involving the infected individual diminishes the infection rate along any edge emanating from him/her by a factor $\alpha _{\mathrm {o}}$. An intervention affecting the susceptible individual decreases, by a factor $\alpha _{\mathrm {i}}$, the infection rate of any edge pointing to him/her. For example, in the case of airborne transmission, a mask may reduce the number of viral particles exhaled by an infected individual and also reduce the number of viral particles that a susceptible can inhale from the environment^20^. As the example of masks shows, the factors $\alpha _{\mathrm {o}}$ and $\alpha _{\mathrm {i}}$ are not necessarily equal. For example valved masks strongly protect the wearer $\alpha _{\mathrm {i}}\ll 1$ while they do not impede viral transmission towards the others $\alpha _{\mathrm {o}}\approx 1$. For cloth masks the opposite is true. We compare scenarios where a single type of PI (with given $\alpha _{\mathrm {o}}$ and $\alpha _{\mathrm {i}}$) is adopted by a fraction *f* of the population. For a given edge pointing from node *i* to node *j*, the infection rate associated to the disease transmission from *i* to *j* takes the form

1 $$\begin{aligned} \beta _{ij} = \beta a_{ij} \left[ 1 - m_i (1- \alpha _{\mathrm {o}}) \right] \left[ 1 - m_j (1- \alpha _{\mathrm {i}})\right] , \end{aligned}$$

where $m_i$ are quenched Bernoulli stochastic variables taking value 1 with probability *f* and zero with probability $1-f$. From this definition it is obvious that, even if the contact network is undirected (symmetric $a_{ij}$)^35^, the transmission network given by (1) is effectively directed, with $\beta _{ij} \ne \beta _{ji}$ unless $f=0$ (no PI adoption in the population) or $f=1$ (whole population adopting PI), which lead to $\beta _{ij} = \beta a_{ij}$ and $\beta _{ij} = \beta \alpha _{\mathrm {i}}\alpha _{\mathrm {o}}a_{ij}$, respectively. In these cases, the infection process is symmetric if the underlying contact network is undirected. In our study we are concerned with the differences between PIs that mostly protect the adopter, characterized by $\alpha _{\mathrm {i}}\ll \alpha _{\mathrm {o}}$, and PIs that mostly protect his/her contacts, given by $\alpha _{\mathrm {o}}\ll \alpha _{\mathrm {i}}$. For the sake of simplicity, we focus on what we call SELF interventions (with $\alpha _{\mathrm {i}}< 1$, $\alpha _{\mathrm {o}}=1$) that offer protection to the adopter and no protection for his/her contacts, and OTHER interventions ($\alpha _{\mathrm {i}}= 1$, $\alpha _{\mathrm {o}}<1$), that offer protection to the contacts, and no protection to the adopter.

In the following, we investigate the effects of PIs in fundamental models of disease propagation, either non-recurrent or recurrent.

### Non-recurrent diseases

As an example of a communicable non-recurrent disease (that is, a disease that confers permanent immunity), we consider the simple Susceptible-Infected-Removed (SIR) model^32^. The SIR model is defined in terms of three compartments. Susceptible individuals are healthy and can contract the disease. Infected individuals carry the disease and can infect susceptible ones upon contact with a rate (probability per unit time) given by the factor $\beta _{ij}$ in Eq. (1). In their turn, infected individuals can recover and become removed with a constant rate $\mu$. By rescaling time, we can absorb the rate $\mu$ and consider a single parameter, the spreading rate $\lambda = \beta / \mu$.

Focusing for simplicity on homogeneous networks with a uniform degree $k_i = K$, a mean-field theoretical analysis (see Methods “Mean-field theory for the SIR model” section) shows that the SIR model exhibits a transition between a phase with non-extensive outbreaks only and a phase with macroscopic ones, at a critical value of the spreading rate $\lambda$ (see Methods “Mean-field theory for the SIR model” section)

2 $$\begin{aligned} \lambda _c = \frac{1}{K-1} \frac{1}{ f \alpha _{\mathrm {i}}\alpha _{\mathrm {o}}+1 - f}. \end{aligned}$$

**Figure 1:**
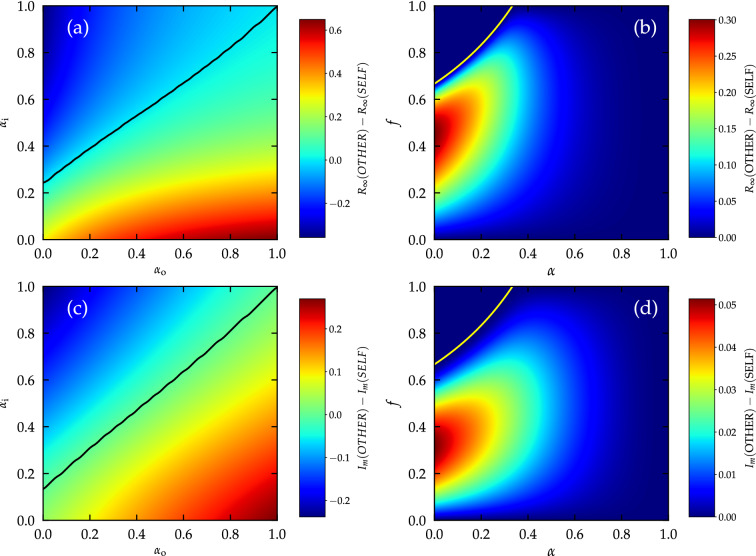
SELF interventions are more effective than OTHER interventions at the population level. (**a**) Difference between the final prevalence $R_\infty$ for the OTHER and the SELF scenario ($R_\infty (\mathrm {OTHER})-R_\infty (\mathrm {SELF})$), as a function of $\alpha _{\mathrm {i}}$ and $\alpha _{\mathrm {o}}$ of the individual PI. Notice that $R_\infty (\mathrm {OTHER})$ depends only on $\alpha _{\mathrm {o}}$, and $R_\infty (\mathrm {SELF})$ depends only on $\alpha _{\mathrm {i}}$. The solid black line indicates the zero value. (**b**) Difference between the final prevalence $R_\infty$ for the OTHER and the SELF scenario as a function of the fraction *f* of adopters and the efficacy $\alpha$ of the individual PI. The solid yellow line denotes where $\lambda _c(\alpha )=\lambda$. Above it, the system is subcritical and $R_\infty (\mathrm {OTHER})-R_\infty (\mathrm {SELF})=0$. (**c**) Difference between the maximum value of the incidence $I_{m}$ for the OTHER and the SELF scenario ($I_m(\mathrm {OTHER})-I_m(\mathrm {SELF})$), as a function of $\alpha _{\mathrm {i}}$ and $\alpha _{\mathrm {o}}$ of the individual PI. Notice that $I_m(\mathrm {OTHER})$ depends only on $\alpha _{\mathrm {o}}$, and $I_m(\mathrm {SELF})$ depends only on $\alpha _{\mathrm {i}}$. The solid black line indicates the zero value. (**d**) Difference between the maximum value of the incidence $I_{m}$ for the OTHER and the SELF scenario as a function of the fraction *f* of adopters and the efficacy $\alpha$ of the individual PI. The solid yellow line denotes where $\lambda _c(\alpha )=\lambda$. In all plots quantities greater than zero indicate that SELF interventions perform better. Solution of homogeneous MF equations for a network of degree $K=7$ and for $\lambda =0.5$. In panels (**a**) and (**c**) $f=0.5$. For these parameters, in panels (**a**) and (**c**) both SELF and OTHER strategies operate above their respective thresholds.

From this expression, we see that the threshold is symmetric in the parameters $\alpha _{\mathrm {i}}$ and $\alpha _{\mathrm {o}}$, in agreement with the results in Ref.^20^, and that the maximum system-wide protection is obtained, for a given value of adoption probability *f*, when $\alpha _{\mathrm {i}}\alpha _{\mathrm {o}}= 0$, which can be attained either in the perfect SELF scenario ($\alpha _{\mathrm {i}}=0$) or in the perfect OTHER case ($\alpha _{\mathrm {o}}=0$). In the same theoretical framework, it is also possible to calculate the total prevalence $R_\infty$, i.e., the overall fraction of individuals infected by the disease throughout the outbreak, obtaining, slightly above the threshold (see Methods “Mean-field theory for the SIR model” section)

3 $$\begin{aligned} R_\infty \simeq \frac{2 \Delta }{\lambda _c^2 (K-1)}\frac{f \alpha _{\mathrm {i}}+1 - f}{f \alpha _{\mathrm {i}}^2 \alpha _{\mathrm {o}}+ 1 - f}, \end{aligned}$$

where $\Delta = \lambda -\lambda _c$ quantifies the distance from the critical threshold. This expression reveals that, at variance with the position of the threshold, the size of an outbreak is not symmetric with respect to the efficacy of PIs: At fixed values of *f*, $\lambda$ and of the threshold $\lambda _c$ (i.e. for the product $\alpha _{\mathrm {i}}\alpha _{\mathrm {o}}$ fixed to a constant *A*), if we substitute $\alpha _{\mathrm {o}}= A / \alpha _{\mathrm {i}}$, the total prevalence is easily shown to be an increasing function of $\alpha _{\mathrm {i}}$, while setting $\alpha _{\mathrm {i}}= A / \alpha _{\mathrm {o}}$ leads to a total prevalence that decreases with $\alpha _{\mathrm {o}}$. In other words, while using more effective SELF interventions (i.e., reducing $\alpha _{\mathrm {i}}$) decreases $R_\infty$, imposing more effective OTHER interventions (i.e reducing $\alpha _{\mathrm {o}}$) creates the opposite result, an increase of $R_\infty$. This signals that SELF interventions, protecting the adopter, are more effective than OTHER interventions, protecting the contacts, at reducing the overall spreading of the disease at the community level. This is confirmed by integrating numerically the homogeneous mean field Eqs. (11)–(15), for generic values of $\alpha _{\mathrm {i}}$ and $\alpha _{\mathrm {o}}$ (see Fig. 1a).

Most of the parameter space results in $R_\infty (\mathrm {OTHER})>R_\infty (\mathrm {SELF})$, i.e. a larger circulation of the disease when OTHER interventions are adopted.

The comparison of SELF and OTHER scenarios provides remarkable insight also at the level of the individual. As shown in Methods “Mean-field theory for the SIR model” section, the relation between the asymptotic prevalence $R_1(\infty )$ for adopting individuals and the prevalence $R_0(\infty )$ for nonadopting ones is

4 $$\begin{aligned} R_1(\infty ) = 1 - [1-R_0(\infty )]^{\alpha _{\mathrm {i}}}, \end{aligned}$$

which does not depend on the protection level $\alpha _{\mathrm {o}}$ offered to contacts. This means that adopting interventions offering more protection to contacts (i.e. reducing $\alpha _{\mathrm {o}}$) has exactly the same effect on the prevalence of adopting and nonadopting individuals. In particular, if OTHER PIs are used, since $\alpha _{\mathrm {i}}=1$ then $R_1(\infty )=R_0(\infty )$, for any value of $\alpha _{\mathrm {o}}$. In such a case, from the point of view of a given individual, adopting an intervention is perfectly equivalent to not adopting it, as the probability to be eventually infected is exactly the same. With the benefit of hindsight this result is not so odd: If $\alpha _{\mathrm {i}}=1$, PIs do not provide any protection against the transmission from others to the adopter, hence for the individual adopter the risk of being infected cannot be smaller than for a non-adopter. However, Eq. (4) reveals that, while adopting a PI always implies some degree of inconvenience (think for example at the bother of wearing a mask), no immediate utility exists for the individual adopting an OTHER PI, while such a utility exists for SELF PIs. See the concluding section for further discussion.

**Figure 2:**
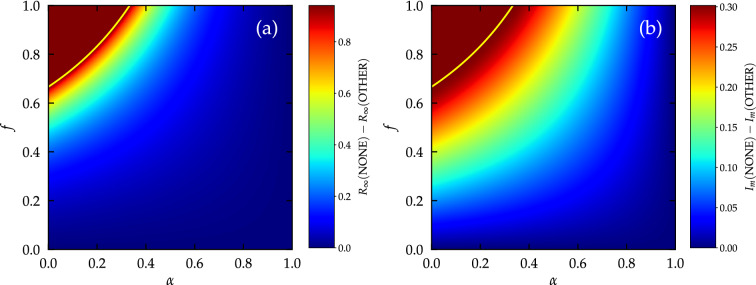
OTHER interventions flatten the curve but barely reduce prevalence with respect to no intervention. (**a**) Difference between the final prevalence *R* for the NONE and the OTHER scenario as a function of the fraction *f* of adopters and the efficacy $\alpha$ of the individual PI. (**b**) Difference between the maximum value of the incidence $I_{m}$ for the NONE and the OTHER scenario as a function of the fraction *f* of adopters and the efficacy $\alpha$ of the individual PI. Solution of MF equations for a homogeneous network of degree $K=7$ for $\lambda =0.5$. The solid yellow lines are where $\lambda _c(\alpha )=\lambda$ for the OTHER scenario.

From now on we mostly focus on the comparison of the effects at the population level of SELF and OTHER interventions with exactly the same efficacy $\alpha$ ($\alpha _{\mathrm {i}}=\alpha$ for the SELF case, $\alpha _{\mathrm {o}}=\alpha$ for the OTHER case). For reference we consider also the case NONE, where no intervention is adopted, corresponding to $\alpha _{\mathrm {i}}= \alpha _{\mathrm {o}}= 1$, and the case BOTH, given by $\alpha _{\mathrm {i}}=\alpha _{\mathrm {o}}=\alpha$, and representing equally protecting performance in both directions. In this last case (i.e., along the diagonal in Fig. 1a) SELF PIs outperform OTHER PIs for any value of the fraction *f* of adopters. This is checked by considering $R_\infty (\mathrm {OTHER})-R_\infty (\mathrm {SELF})$ as a function of $\alpha$ and *f*. The values plotted in Fig. 1b, which are nonnegative for any value of $\alpha$ and *f*, demonstrate that in all cases SELF interventions are better than or equal to OTHER interventions in curbing disease diffusion. These conclusions remain true for other values of the infectivity $\lambda >\lambda _c$ as we can see from Supplementary Figs. SF1 to SF4, where we present figures analogous to Figs. 1 and 2 for $\lambda = 1.0$ and $\lambda =2.0$. Interestingly, from Fig. 1b it turns out that the difference between the two types of PI is maximal for very small values of $\alpha$ (as expected) and in general, for intermediate, $\alpha$-dependent values of the adoption fraction *f*.

The numerical solution of MF equations also allows us to determine the height $I_m$ of the peak of incidence over time. This is a crucial quantity that must be kept as low as possible in order to avoid the saturation of health care systems by an inflow of too many seriously ill individuals. Figure 1c shows clearly that also in this respect SELF PIs perform better than the OTHER counterparts, as witnessed by the asymmetry of the plot with respect to the diagonal. The same conclusion is confirmed by Fig. 1d: $I_m(\mathrm {OTHER})-I_m(\mathrm {SELF})\ge 0$ for any value of $\alpha$ and *f*. Adopting SELF interventions “flattens the curve” more effectively than the adoption of OTHER PIs does. A comparison between panels (b) and (d) in Fig. 1 reveals also that PI adoption affects differently the total number of infected individuals with respect to the peak incidence. In particular, the improved performance of SELF PIs is maximized for $\alpha \rightarrow 0$ and $f\approx 0.3$ for what concerns the flattening of the curve, while $f\approx 0.45$ for the total size of the outbreak.

Unanticipated effects can also be observed by comparing the performance of OTHER PIs with a scenario with no interventions at all, and given by $\alpha _{\mathrm {i}}= \alpha _{\mathrm {o}}= 1$, see Fig. 2. While it is expected that the effect of PIs grows with efficacy (small $\alpha$) and widespread PI adoption (*f* close to 1) it is surprising to see that in a large domain of the parameter space $R_\infty (\mathrm {NONE})-R_\infty (\mathrm {OTHER})$ is very close to zero (PIs do not significantly change the number of people eventually infected) while $I_m(\mathrm {NONE})-I_m(\mathrm {OTHER})$ is rather large: OTHER PIs do not lead to an overall reduction of the outbreak size but indeed substantially flatten the curve.

**Figure 3:**
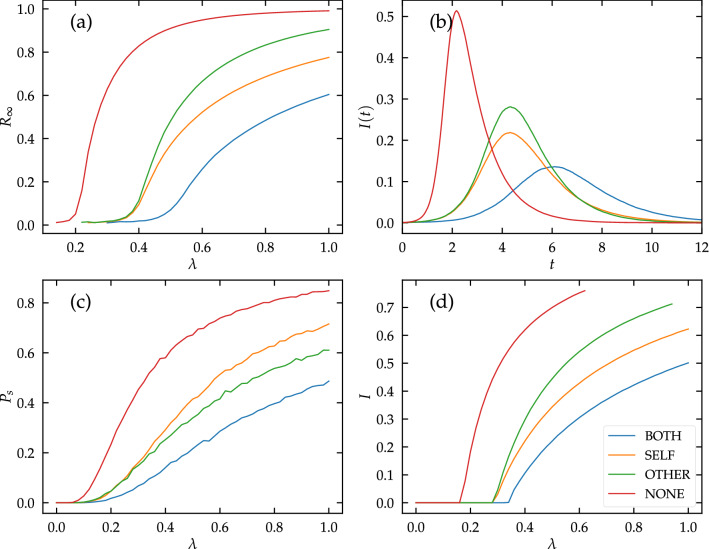
Numerical simulations confirm analytical results. Results of numerical simulations on a random regular network of degree $K=7$. The various curves are for the different scenarios of PI deployment. In all cases $f=0.5$ and $\alpha =0.2$. SIR dynamics: (**a**) Value of the average size $R_\infty$ of extensive outbreaks as a function of $\lambda$; network size $N=10^4$, single random infected seed. (**b**) Temporal evolution of the incidence *I*(*t*) for a single realization of the dynamics; network size $N=10^5$, 50 initial random infected seeds, $\lambda =0.5$. The exponential growth is the same for SELF and OTHER scenarios, but it lasts longer in the OTHER case. (**c**) Value of the probability $P_s$ to observe an extensive outbreak as a function of $\lambda$; network size $N=10^3$, single random infected seed. SIS dynamics: (**d**) Value of the stationary density *I* of infected nodes as a function of $\lambda$. Network size $N=10^4$.

The previous predictions have been obtained within the mean-field framework. The comparison with numerical simulations, to check whether the picture derived above remains true, requires to take into account the phenomenology of the stochastic SIR model in finite systems (see Methods “Phenomenology of the SIR model in finite networks” section), but reveals new interesting features. Figure 3a shows that, also in numerical simulations, the better efficacy of SELF interventions at the population level remains valid for any value of the infectivity $\lambda$. Indeed the average size $R_\infty$ of extensive outbreaks is smaller when SELF PIs, rather than OTHER PIs, are adopted.

Different values of the asymptotic prevalence for long times $R_\infty$ reflect a different temporal evolution. The mean-field approach predicts (see Methods “Mean-field theory for the SIR model” section) that the initial growth is exponential, with a characteristic time scale depending only on the threshold value, and thus on the product $\alpha _{\mathrm {o}}\alpha _{\mathrm {i}}$, and hence is equal for both SELF and OTHER scenarios with the same $\alpha _{\mathrm {i}}=\alpha _{\mathrm {o}}=\alpha$. The difference in the value of $R_\infty$ stems from the fact that this exponential growth ends later in the OTHER case, thus corresponding to a higher value of the peak incidence $I_{m}$. Notice that this is different with what happens for the NONE ($\alpha _{\mathrm {i}}=1$, $\alpha _{\mathrm {o}}=1$) or BOTH ($\alpha _{\mathrm {i}}=\alpha _{\mathrm {o}}<1$) scenarios. In these other cases already the exponential growth rate is different. Performing simulations of the stochastic SIR model (see Methods “Phenomenology of the SIR model in finite networks” section) and analyzing the temporal evolution of the number of infected individuals (see Fig. 3b), these differences are clearly observed. The maximum value of the incidence in the SELF case is in general lower than in the OTHER case: adopter-protecting interventions are more effective than contact-protecting interventions in “flattening the curve” and thus reduce the maximum instantaneous pressure on health care systems.

SELF interventions, however, are not the silver bullet against epidemics. Indeed, while they reduce the overall prevalence of the infectious disease, they are worse than OTHER interventions at preventing the emergence of a macroscopic outbreak. As Fig. 3c shows, the probability that a macroscopic outbreak emerges is larger for the SELF prescription than for the OTHER one. This means that, if people adopt SELF PIs, it is more likely that a single infected individual can give rise to a macroscopic outbreak. However, as shown above, if such an outbreak occurs, or if the disease is introduced in different nodes (for example because several infected individuals arrive simultaneously in a community) the outbreak will affect, on average, less people if SELF PIs are adopted.

### Recurrent diseases

The paradigmatic model for recurrent diseases, leading to a steady endemic state, is the Susceptible-Infected-Susceptible (SIS) model^32^. In this case the epidemic transition separates values of the parameter $\lambda =\beta /\mu$ for which the disease quickly goes extinct ($\lambda \le \lambda _c$), from values such that an endemic state is reached ($\lambda >\lambda _c$), with a steady fraction of infected individuals. Also in this case it is possible to investigate the effect of different types of interventions via a mean-field approach for homogeneous networks (see Methods “Mean-field theory for the SIS model” section), obtaning for the threshold

5 $$\begin{aligned} \lambda _c = \frac{1}{K} \frac{1}{f \alpha _{\mathrm {i}}\alpha _{\mathrm {o}}+ 1 - f}, \end{aligned}$$

while the stationary density of infected nodes in the vicinity of this threshold is

6 $$\begin{aligned} I \simeq \frac{\Delta }{\lambda _c^2 K} \frac{f \alpha _{\mathrm {i}}+ 1 - f}{f \alpha _{\mathrm {o}}\alpha _{\mathrm {i}}^2 +1 -f}. \end{aligned}$$

Hence the phenomenology of the SIS model is analogous to what is found for SIR: The onset of the endemic state is a symmetric function of the PI efficacies. The prevalence about it, however, is, for a constant $\alpha _{\mathrm {o}}\alpha _{\mathrm {i}}$, an increasing function of $\alpha _{\mathrm {i}}$ and a decreasing one of $\alpha _{\mathrm {o}}$. Interventions protecting the adopter (small $\alpha _{\mathrm {i}}$, $\alpha _{\mathrm {o}}=1$) result in lower disease circulation at the population level than interventions protecting contacts of the adopter (small $\alpha _{\mathrm {o}}$, $\alpha _{\mathrm {i}}=1$). These predictions are verified numerically to be true for all values of $\lambda >\lambda _c$, not only close to the transition (see Fig. 3d).

### An application to COVID-19 diffusion

While we have considered so far extremely simple epidemic models on idealized networks, the qualitative conclusions extend to more complicated epidemic models and more general types of networks. For example, the extension to heterogeneous networks with a general degree distribution *P*(*k*), presented in the Supplementary Information, leads to results perfectly analogous to those discussed above. To give a more realistic example, here we consider a Susceptible-Exposed-Infected-Recovered (SEIR) dynamics on a large weighted network describing interactions among people in Portland, OR^36^. This network has been inferred using mobile device data before social distancing measures were enacted during the COVID-19 pandemic. SEIR model parameters were calibrated to describe the first wave of COVID-19 in 2020 (see Methods “Details on the network and the epidemic model for COVID-19” section for more details). The size of the network is $N=214,393$, which is a substantial fraction of the census population of Portland city in 2020, namely $N_P = 652,503$^37^.

**Figure 4:**
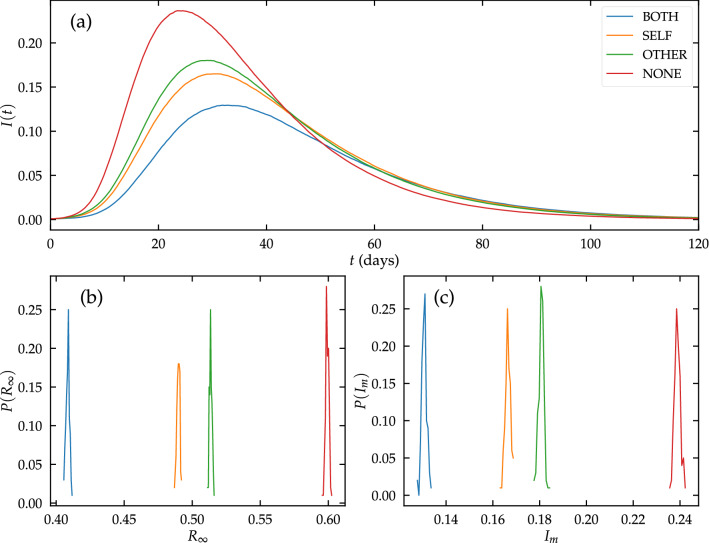
SEIR dynamics on the Portland network. (**a**) *I* vs *t* for a single run; (**b**) Distribution of the fraction *R* of recovered people in the asymptotic state for 100 runs in each of the 4 scenarios; (**c**) Distribution of the maximum fraction $I_m$ of simultaneously infected people for 100 runs in each of the 4 scenarios.

In this framework, we tested the four scenarios for PI efficacy, assuming again $f=0.5$. Figure 4 shows that also in this case SELF interventions flatten the curve of the number of infected people more effectively than OTHER interventions with the same individual efficacy. With respect to the final prevalence $R_\infty$, adopting SELF PI reduces the value in the absence of interventions $R_\infty (\mathrm {NONE})= 0.595$ to $R_\infty (\mathrm {SELF})=0.487$, which is significantly (beyond stochastic fluctuations) smaller than $R_\infty (\mathrm {OTHER})=0.510$, while for interventions with both SELF and OTHER efficacy the prevalence is $R_\infty (\mathrm {BOTH})=0.406$. On the scale of the city, extrapolating these values for a population of $N_P = 652,503$, the adoption of SELF PI instead of OTHER PI would imply a reduction of around 15, 000 in the total number of cases. For the maximum number of simultaneously infected people, we obtain the values $I_m(\mathrm {NONE})= 0.238$, $I_m(\mathrm {SELF})= 0.166$, $I_m(\mathrm {OTHER})= 0.180$, and $I_m(\mathrm {BOTH})= 0.130$, which on the city scale implies a reduction of around 9, 000 concurrently infected individuals when SELF interventions are used instead of OTHER interventions.

Although we do not claim these numbers to be directly applicable to a real world scenario, the results clearly show that the collective effects leading to a better performance of SELF interventions with respect to OTHER interventions at the population level are at work also for more complicated epidemic models on realistic networks.

## Discussion

A vast range of protecting interventions of different type, both pharmaceutical and non-pharmaceutical, can be used to combat the diffusion of an infectious disease. Each intervention may in general affect differently the infectivity and the susceptibility of the single individual.

Here we have considered, in a simple formalism based on disease propagation on networks, the effects of generic PIs on the reduction of the total number of infected individuals and of the maximum number of individuals concurrently infected, the so-called goal of “flattening the curve”, to prevent the saturation of health care systems. In the simple setting adopted, PIs are characterized by two parameters which, in the case of masks, gauge the level of viral particle filtration offered from the mask wearer to the environment ($\alpha _{\mathrm {o}}$), and in the opposite direction ($\alpha _{\mathrm {i}}$).

The results presented above demonstrate that collective effects have surprising consequences for what concerns the level of global protection guaranteed by different kinds of intervention. In agreement with many other publications, we find, of course, that no matter how imperfect, the adoption of PIs reduces the circulation of an infectious disease with respect to the case of no adoption, and that a higher efficiency at the individual level guarantees a higher protection for the whole community. Surprisingly, however, we find that interventions protecting the adopter (SELF PIs) or interventions protecting mostly contacts of the adopter (OTHER PIs) have *not* the same effect, even for equal efficacy of the individual intervention. SELF PIs turn out to reduce more the overall incidence of the infection and to “flatten the curve” more efficiently, i.e., they reduce more drastically the peak value of the number of individuals simultaneously infected. Although we have focused on purely SELF ($\alpha _{\mathrm {o}}=1$, $\alpha _{\mathrm {i}}<1$) and purely OTHER ($\alpha _{\mathrm {o}}<1$, $\alpha _{\mathrm {i}}=1$) interventions, the same picture applies when SELF (OTHER) PIs provide some small level of protection also in the other direction, i.e. $\alpha _{\mathrm {i}}\ll \alpha _{\mathrm {o}}$ ($\alpha _{\mathrm {i}}\gg \alpha _{\mathrm {o}}$), respectively. Our results are presented in terms of a mean-field theory for the simple SIR and SIS models of disease propagation in homogeneous networks, that can be readily extended to heterogeneous contact patterns (see Supplementary Information), and are backed up with numerical simulations. Additionally, we consider a SEIR model, often used for COVID-19 analyses and estimations, on an empirical network representing contact patterns in the city of Portland. This last analysis allows us to confirm the expected effect of the different kinds of PIs in a realistic scenario. Our results can be interpreted in the framework of the general theory for heterogeneous agents put forward by Miller^34^, predicting that control strategies having a heterogeneous impact on susceptibilities are more effective in reducing epidemic size while strategies with heterogeneous impact on infectivities reduce more the probability of large outbreaks.

What causes the difference between the performance of the SELF and OTHER strategies? To investigate this issue we considered a system where the choice of the PI is not individual-based, but contact-based: in other words, $\beta _{ij}$ is still a quenched random variable given by (1), but the variables $m_i$ and $m_j$ are extracted independently for each edge. Hence, considering the example of mask wearing, an individual *i* can use a mask when interacting with contact $j_1$ and not use it when interacting with contact $j_2$. In such a case (see Supplementary Fig. SF5) SELF and OTHER scenarios give exactly the same results. Therefore asymmetry arises at the community level only if each individual consistently adopts (or does not adopt) the protecting intervention in every interaction.

Concerning the example of masks, our results place a strong question mark on policies adopted to curb the diffusion of COVID-19. During the initial stages of the pandemic, concerns were raised about the use of valved masks, as it was argued that they do not protect contacts^38^. This led to advice against their use by the World Health Organization (WHO)^39^ and the Centers for Disease Control and Prevention (CDC)^40^, and to outright bans in some cities^41^ and in major US airlines^42^. At the same time the usage of masks protecting contacts of the wearer has been encouraged. As we have shown, unless effective masks providing protection in both directions can be widely used, it is better to adopt SELF (valved) masks to maximally reduce the impact of the disease at the population level.

This global effect is not the only argument pushing to reconsider this type of policies. Another compelling reason has to do with the utility of single individuals. The usage of PIs can be imposed by ordinances issued by the government, but the adoption of individual PIs, even if policed by authorities, always involves a personal choice. As shown above, the adoption of an intervention that protects only the contacts of the adopter ($\alpha _{\mathrm {i}}=1$) does not imply any direct utility for the adopter: The probability to be eventually infected is exactly the same for adopting and nonadopting persons. On the other hand, adopting an intervention (for example wearing a mask) is a burden, so that the net direct utility for adopting an OTHER PI is negative. A positive overall payoff for the individual adopting an OTHER PI can be obtained only if many people cooperate so that the common good is achieved, overcoming the inconvenience implied by the adoption. Thus OTHER PIs are both less effective at the population level and less likely to be adopted at the individual one. No such barrier to adoption exists for self-protecting (SELF) PIs: In such a case it is not only beneficial at the collective level, but also in the selfish interest of each individual to adopt a protecting intervention. Hence, we conjecture that the overall higher effectiveness of self-protecting interventions at the community level will be further enhanced by the fact that they are more likely to be spontaneously adopted.

## Methods

### Mean-field theory for the SIR model

In the case of a homogeneous network, with all nodes sharing the same degree $k = K$, a mean-field approximation considers that all nodes with the same PI state are equivalent, and therefore we can characterize the dynamics by the probability that a node in a given PI state (adopter, nonadopter) is in the compartment *S*, *I*, or *R*. Under these conditions, the dynamics of the network is defined by the set of probabilities

7 $$\begin{aligned} I_a, \quad S_a, \quad R_a, \end{aligned}$$

that an individual in the PI state *a*, taking values $a=1$ (adopter) and $a = 0$ (nonadopter), is infected, susceptible or recovered, respectively. In a population of fixed size, these sets of probabilities are subject to the normalization condition

8 $$\begin{aligned} I_a + S_a + R_a = 1. \end{aligned}$$

The mean-field equations for the system are constructed by considering the contacts that can change the number of infected individuals in each PI state. Thus, the rate equation for the number of removed individuals takes the form

9 $$\begin{aligned} \frac{d R_a}{d t} = \mu I_a, \end{aligned}$$

for all PI states. For the density of infected adopting individuals, we can write^30,32^

10 $$\begin{aligned} \frac{d I_1}{d t} =-\mu I_1 + (K-1) S_1 \left[ f I_1 \beta \alpha _{\mathrm {i}}\alpha _{\mathrm {o}}+ (1-f) I_0 \beta \alpha _{\mathrm {i}}\right] . \end{aligned}$$

In this equation, the first term considers the decay to the recovered state with rate $\mu$. The second term considers the probability of a new infection along a given edge, that is proportional to the density of susceptible adopting individuals, and the probability that the edge points to an adopting infected individual (with probability *f*), which will lead to an effective infection rate $\beta \alpha _{\mathrm {i}}\alpha _{\mathrm {o}}$, or to an infected nonadopting individual (with probability $1-f$), associated to an effective infection rate $\beta \alpha _{\mathrm {i}}$. The term $K-1$ takes into account that infected individuals have at most only $K-1$ edges available to propagate the infection, since one is used up by the neighbor that induced their infection. Rescaling time by $\mu$, we can write

11 $$\begin{aligned} \frac{d I_1}{d t} = - I_1 + \lambda \alpha _{\mathrm {i}}(K-1) S_1 \theta , \end{aligned}$$

where $\lambda = \beta /\mu$ and we have defined

12 $$\begin{aligned} \theta =f \alpha _{\mathrm {o}}I_1 + (1-f) I_0. \end{aligned}$$

From here, the rate equation for adopting susceptible individuals is

13 $$\begin{aligned} \frac{d S_1}{d t} = - \lambda \alpha _{\mathrm {i}}(K-1) S_1 \theta . \end{aligned}$$

Finally, for nonadopting individuals, we can write

14 $$\begin{aligned} \frac{d I_0}{d t}= & {} -I_0 + \lambda (K-1) S_0 \theta , \end{aligned}$$

15 $$\begin{aligned} \frac{d S_0}{d t}= & {} -\lambda (K-1) S_0 \theta . \end{aligned}$$

From the general Eq. (9), considering that $\mu$ is absorbed into the time rescaling, we have

16 $$\begin{aligned} R_a(t) = \int _0^t I_a(t') dt', \end{aligned}$$

and from Eqs. (13) and (15),

17 $$\begin{aligned} S_1(t) = e^{- \lambda (K-1) \alpha _{\mathrm {i}}\phi (t)}, \quad S_0(t) = e^{ - \lambda (K-1) \phi (t)}, \end{aligned}$$

where $\phi (t) = \int _0^t \theta (t') dt'$ can be written as

18 $$\begin{aligned} \phi (t) = f \alpha _{\mathrm {o}}R_1(t) + (1-f) R_0(t), \end{aligned}$$

and where we assume an initial condition given by a vanishing fraction of infected individuals.

#### Threshold evaluation

To estimate the value of the threshold, we consider the final outbreak size, given by the number of removed individuals at time $t \rightarrow \infty$. Since $I_a(\infty ) = 0$, we have from the normalization condition, Eq. (8), $R_a(\infty ) = 1 - S_a(\infty )$. In this infinite time limit, defining $\phi _\infty \equiv \phi (\infty )$,

19 $$\begin{aligned} \phi _\infty= & {} f \alpha _{\mathrm {o}}R_1(\infty ) + (1-f) R_0(\infty ) \end{aligned}$$

20 $$\begin{aligned}= & {} f \alpha _{\mathrm {o}}[1 - e^{- \lambda (K-1) \alpha _{\mathrm {i}}\phi _\infty }] + (1-f) [1 - e^{- \lambda (K-1) \phi _\infty }] \equiv \Psi (\phi _\infty ). \end{aligned}$$

A non-zero solution is obtained when $\left. \frac{d \Psi (\phi _\infty )}{ d \phi _\infty }\right| _{\phi _\infty = 0} \ge 1$, leading to the threshold condition $\lambda > \lambda _c$ to observe a finite outbreak, with

21 $$\begin{aligned} \lambda _c = \frac{1}{(K-1) \left[ f \alpha _{\mathrm {i}}\alpha _{\mathrm {o}}+1 - f \right] }. \end{aligned}$$

#### Behavior close to the threshold

In the limit $\lambda \rightarrow \lambda _c^+$, we have that $R_a(\infty )$ and $\phi _\infty$ are both small. From Eq. (19), performing an expansion for small $\phi _\infty$ up to second order, we can solve it and obtain

22 $$\begin{aligned} \phi _\infty \simeq \frac{2 \Delta }{\lambda _c^3 (K-1)^2} \frac{1}{f \alpha _{\mathrm {o}}\alpha _{\mathrm {i}}^2 +1 - f}, \end{aligned}$$

where $\Delta = \lambda - \lambda _c$ is the distance to the critical threshold. For the total prevalence, $R_\infty = f R_1(\infty ) + (1-f)R_0(\infty )$, using Eq. (17) and the normalization condition, we can write

23 $$\begin{aligned} R_\infty= & {} f [1 - e^{- \lambda (K-1) \alpha _{\mathrm {i}}\phi _\infty }] + (1-f) [1 - e^{- \lambda (K-1) \phi _\infty }] \nonumber \\\simeq & {} \lambda (K-1) [f \alpha _{\mathrm {i}}+1 - f] \phi _\infty , \end{aligned}$$

where the have expanded the exponential factors to leading order. Combining Eqs. (23) and (22), we finally obtain the total prevalence close to the threshold

24 $$\begin{aligned} R_\infty \simeq \frac{2 \Delta }{\lambda _c^2 (K-1)}\frac{f \alpha _{\mathrm {i}}+1 - f}{f \alpha _{\mathrm {i}}^2 \alpha _{\mathrm {o}}+ 1 - f}. \end{aligned}$$

#### Relation between $R_0(\infty )$ and $R_1(\infty )$

Taking the limit $t \rightarrow \infty$ in (17) and eliminating $\phi _\infty$ we obtain $S_1 = S_0^{\alpha _{\mathrm {i}}}$, from which, using the condition $R_a(\infty ) = 1 - S_a(\infty )$, we arrive at

25 $$\begin{aligned} R_1(\infty ) = 1 - [1-R_0(\infty )]^{\alpha _{\mathrm {i}}}. \end{aligned}$$

#### Initial time behavior

To estimate the initial time behavior of the epidemic outbreak, we consider the limit of a very small density of infected individuals. Linearizing the corresponding equations, we have

26 $$\begin{aligned} \dot{I}_1\simeq & {} - I_1 + \lambda (K-1) \alpha _{\mathrm {i}}\theta , \end{aligned}$$

27 $$\begin{aligned} \dot{I}_0\simeq & {} - I_0 + \lambda (K-1) \theta . \end{aligned}$$

Using these expressions in Eq. (12), we have

28 $$\begin{aligned} \dot{\theta } = f \alpha _{\mathrm {o}}\dot{I}_1 + (1-f)\dot{I}_0 \simeq -\theta + \frac{\lambda }{\lambda _c}\theta . \end{aligned}$$

Defining the characteristic time scale

29 $$\begin{aligned} \tau = \frac{\lambda _c}{\lambda - \lambda _c}, \end{aligned}$$

the solution for $\theta$ is

30 $$\begin{aligned} \theta (t) = \theta (0) e^{t/\tau }. \end{aligned}$$

Using these expressions, we can integrate Eqs. (26) and (27) to obtain

31 $$\begin{aligned} I_1(t)\simeq & {} \lambda _c(K-1) \alpha _{\mathrm {i}}\theta (0) e^{t/\tau } \end{aligned}$$

32 $$\begin{aligned} I_0(t)\simeq & {} \lambda _c(K-1) \theta (0) e^{t/\tau }, \end{aligned}$$

while the total incidence $I(t) = f I_1(t) + (1-f) I_0(t)$ has the form

33 $$\begin{aligned} I(t) \simeq \lambda _c (K-1) \left[ f \alpha _{\mathrm {i}}+ 1 - f \right] \theta (0) e^{t/\tau }. \end{aligned}$$

For the initial condition, let us consider that a randomly chosen node is initially infected. In this case, $I_m(0) = I_0(0) = 1/N$, and therefore

34 $$\begin{aligned} \theta (0) = \frac{1}{N} \left[ f \alpha _{\mathrm {o}}+ 1 -f \right] . \end{aligned}$$

Therefore

35 $$\begin{aligned} I(t) \simeq \frac{\lambda _c (K-1)}{N} \left[ f \alpha _{\mathrm {i}}+ 1 - f \right] \left[ f \alpha _{\mathrm {o}}+ 1 -f \right] e^{t/\tau }. \end{aligned}$$

### Phenomenology of the SIR model in finite networks

#### Two types of outbreaks

Starting from a single infected node, outbreaks can develop in two qualitatively different ways^43^. Some of them die after a few infection events; others instead survive much longer and affect an extensive fraction of the individuals. This is reflected in the distribution of outbreak sizes, which has two components. For small values of $\lambda$ only the small component exists, made up by short-lived small outbreaks and weakly depending on *N*. For large values of $\lambda$ also the second component exists, peaked around a size proportional to *N*. The epidemic threshold marks the birth of the large, extensive component. This distinction is exactly defined only in the thermodynamic limit (indeed the threshold is properly defined only in this limit), but it is operatively meaningful also for finite, but large, size *N*. The fraction of outbreaks belonging to the large component is the probability $P_s$ that an extensive outbreak emerges. It is zero below the threshold and grows with $\lambda >\lambda _c$ above it. The average size of extensive outbreaks $R_\infty$ (i.e. the average value calculated only for the large component) is another observable that is zero up to $\lambda _c$ and grows with $\lambda$. For epidemics on undirected networks the two quantities coincide^43^. On directed networks (such as the effective networks over which the epidemic spreads if PIs are used) they are in general different^34,44^.

By construction, the mean-field approach on networks^30,43^ deals only with extensive outbreaks in an infinite size system. Hence the quantity $R_\infty$ computed above has to be compared with the average size of macroscopic outbreaks, which must be defined, in simulations, as outbreaks larger than a given fraction of the whole system. We take this fraction to be 0.01. Clearly only for $N \rightarrow \infty$ and sufficiently far from the transition point, results are independent from the choice of this fraction.

#### Temporal evolution

For simulations in finite systems starting with a single infected node, one must take into account that the exponential growth is preceded by a regime dominated by stochastic fluctuations, whose duration has quite large variations depending on the realization of the process. Only when a sufficiently large fluctuation in the number of infected nodes *I*(*t*) is generated, exponential growth is triggered. Averaging at fixed time the value of *I*(*t*) over many such realizations leads to a spurious bending as a function of time. Moreover, the duration of the initial stochastic regime (and hence the size of the spurious bending) is different in the various scenarios, further complicating the analysis. To overcome these difficulties, as it is customary, we start from a larger number of initial infected nodes, namely 50, so that the initial regime dominated by fluctuations is strongly suppressed.

### Mean-field theory for the SIS model

The mean-field equations for the SIS model in a homogeneous network of degree $k_i = K$ depend only on the densities of infected adopting and nonadopting individuals $I_a$, and take the form^30,32^

36 $$\begin{aligned} \dot{I_1}= & {} - I_1 + (1 - I_1) \lambda K \alpha _{\mathrm {i}}\theta \end{aligned}$$

37 $$\begin{aligned} \dot{I_0}= & {} - I_0 + (1 - I_0) \lambda K \theta , \end{aligned}$$

with

38 $$\begin{aligned} \theta = f \alpha _{\mathrm {o}}I_1 + (1-f)I_0. \end{aligned}$$

#### Threshold evaluation

We can compute the threshold $\lambda _c$ by performing a linear stability analysis around the solution $I_a = 0$, corresponding to the healthy, non-endemic state. The Jacobian matrix of Eqs. (36) and (37), evaluated at the origin, is

39 $$\begin{aligned} J = \begin{pmatrix} -1 + \lambda K f \alpha _{\mathrm {i}}\alpha _{\mathrm {o}}&{}\quad \lambda K (1-f) \alpha _{\mathrm {i}}\\ \lambda K f \alpha _{\mathrm {o}}&{}\quad \lambda K (1-f) \end{pmatrix}, \end{aligned}$$

whose associated eigenvalues are $\Lambda _1 = -1$ and $\Lambda _2 = -1 + \lambda K (f \alpha _{\mathrm {i}}\alpha _{\mathrm {o}}+1 - f)$. The healthy state becomes unstable when the largest eigenvalue becomes positive, that is, $-1 + \lambda K (f \alpha _{\mathrm {i}}\alpha _{\mathrm {o}}+1 - f) > 0$. This defines the threshold $\lambda _c$, given by

40 $$\begin{aligned} \lambda _c = \frac{1}{K}\frac{1}{f \alpha _{\mathrm {i}}\alpha _{\mathrm {o}}+1 - f}, \end{aligned}$$

such that for $\lambda > \lambda _c$ there is a steady infected state with a non-zero prevalence.

#### Behavior close to the threshold

For homogeneous networks of degree *K*, the steady state condition $\dot{I}_a = 0$ translates into the relations

41 $$\begin{aligned} I_1= & {} \frac{\lambda K \alpha _{\mathrm {i}}\theta }{1 + \lambda K \alpha _{\mathrm {i}}\theta }, \end{aligned}$$

42 $$\begin{aligned} I_0= & {} \frac{\lambda K \theta }{1 + \lambda K \theta }. \end{aligned}$$

Inserting these into the definition of $\theta$, Eq. (38), we obtain the self-consistent equation

43 $$\begin{aligned} \theta = \frac{\lambda K f \alpha _{\mathrm {i}}\alpha _{\mathrm {o}}\theta }{1 + \lambda K \alpha _{\mathrm {i}}\theta } + \frac{\lambda K (1-f) \theta }{1 + \lambda K \theta }. \end{aligned}$$

Equation (43) can be solved close to the threshold, in the limit of small $\theta$, performing a Taylor expansion up to second order, which leads to the solution

44 $$\begin{aligned} \theta \simeq \frac{\Delta }{\lambda _c^3 K^2} \frac{1}{f \alpha _{\mathrm {o}}\alpha _{\mathrm {i}}^2 +1 -f}. \end{aligned}$$

Using the lowest order approximation $I_1 \simeq \lambda K \alpha _{\mathrm {i}}\theta$ and $I_0 \simeq \lambda K \theta$, the final steady state prevalence $I = f I_1 + (1-f) I_0$ takes the form, close to the threshold

45 $$\begin{aligned} I \simeq \frac{\Delta }{\lambda _c^2 K} \frac{f \alpha _{\mathrm {i}}+ 1 - f}{f \alpha _{\mathrm {o}}\alpha _{\mathrm {i}}^2 +1 -f}, \end{aligned}$$

in close analogy to the SIR result in Eq. (24).

#### Initial time behavior

It is easy to see that the initial time behavior of the SIS model is described by the same equations as the SIR case, simply replacing the factor $K-1$ by *K*. Therefore, the time initial time evolution of an outbreak initiated by a randomly chosen node takes the form, as Eq. (35),

46 $$\begin{aligned} I(t) \simeq \frac{\lambda _c K}{N} \left[ f \alpha _{\mathrm {i}}+ 1 - f \right] \left[ f \alpha _{\mathrm {o}}+ 1 -f \right] e^{t/\tau }. \end{aligned}$$

### Details on the network and the epidemic model for COVID-19

We consider as the contact pattern for the SEIR dynamics the static contact network determined in Ref.^36^ for the city of Portland, Oregon, before social distancing measures were enacted. The network has $N=$ 214,393 nodes and $M=$ 1,538,092 edges, so that the average degree is $\left\langle {k} \right\rangle =14.4$. Further details on network statistics can be found in the original publication. The network is undirected and weighted, with each contact weighted according to its duration.

On top of this network we perform simulations of the SEIR epidemic model in continuous time, using the Gillespie algorithm. The model is characterized by three parameters. An exposed node E spontaneously becomes infectious (I) at a rate $\alpha$, while the transition between the infectious state I to the recovered state R occurs spontaneously at rate $\mu$. In the absence of PI, an infectious node *i* transmits the infection to a susceptible neighbor *j* at a rate $\beta w_{ij}$ where $w_{ij}$ is the weight associated to the network edge. In the presence of PIs this quantity is further modified as in Eq. (1). The values of the parameters are the same calibrated for COVID-19 in Ref.^36^: $\alpha =1/3$, $\nu =1/14$, $\beta =1.337$. The initial condition for simulations is that a fraction $10^{-3}$ of the individuals is infected and the rest is susceptible.

## Supplementary Information

Supplementary Information.

## Data Availability

The datasets used and/or analysed during the current study are available from the corresponding author on reasonable request.
