# Supplementary material for: The advantage of self-protecting interventions in mitigating epidemic circulation at the community level
Source: Sci Rep. 2022 Sep 24;12:15950. doi: 10.1038/s41598-022-20152-4 (PMC9509388; doi:10.1038/s41598-022-20152-4)
Supplement: Supplementary file 1 — Supplementary Information. [file 41598_2022_20152_MOESM1_ESM.pdf]

The advantage of self-protecting interventions in mitigating epidemic circulation at the community level
Supplementary Information

Romualdo Pastor-Satorras[1] and Claudio Castellano[2,3]

[1] *Departament de Física, Universitat Politècnica de Catalunya, Campus Nord B4, 08034 Barcelona, Spain*
[2] *Istituto dei Sistemi Complessi (ISC-CNR), Via dei Taurini 19, I-00185 Rome, Italy*
[3] *Centro Ricerche Enrico Fermi, Piazza del Viminale, 1, I-00184 Rome, Italy*

I. HETEROGENEOUS MEAN-FIELD THEORY FOR THE SIR MODEL

In the case of heterogeneous networks with a non-trivial degree distribution, heterogeneous mean-field (HMF) theory [1] considers that all nodes in the network with the same degree share the same dynamical state, and therefore we can characterize the state of the epidemics in terms of the probability that a node of degree k is in the state S, I, or R, taking the same value for all nodes with the same degree. In the case of a population with a fraction f of protecting intervention (PI) adopters we must consider the independent probabilities for adopting and nonadopting individuals, in such a way that the state of the network is fully described by the set of probabilities

$$I_k^1, \quad S_k^1, \quad R_k^1 \tag{1}$$

that an adopting individual of degree k is infected, susceptible or recovered, respectively, and the corresponding probabilities for nonadopting individuals

$$I_k^0, \quad S_k^0, \quad R_k^0. \tag{2}$$

Both sets of probabilities are subject to the normalization condition

$$I_k^1 + S_k^1 + R_k^1 = I_k^0 + S_k^0 + R_k^0 = 1. \tag{3}$$

In order to construct the rate equations for the time evolution of these variables we need to consider the probability that a randomly chosen link points to an infected node. From a mean field point of view, considering that any individual has a probability f to adopt a PI, and assuming that the contact networks are undirected and uncorrelated [2, 3] we can write the rate equation for the density of infected adopting individuals of degree k as [1, 4, 5]

$$\frac{dI_k^1}{dt} = -\mu I_k^1 + S_k^1 k \sum_{k'} \frac{(k'-1)P(k')}{\langle k \rangle} \left[f I_{k'}^1 \beta \alpha_\mathrm{i} \alpha_\mathrm{o} + (1-f) I_{k'}^0 \beta \alpha_\mathrm{i} \right]. \tag{4}$$

In this equation, the second factor considers the probability that a link emanating from any node points to a node of degree k' (taking into account in the -1 term that an infected node is necessarily connected to at least another infected node that transmitted the disease to it and is thus not capable of further transmission), and the probability that this node of degree k' either adopts a PI (with probability f) and the effective infection rate is $\beta\alpha_\mathrm{i}\alpha_\mathrm{o}$, or he/she does not adopt a PI (with probability $1-f$), with an effective infection rate $\beta\alpha_\mathrm{i}$. Performing a time rescaling $t \to t\mu$, Eq. (4) can be written as

$$\frac{dI_k^1}{dt} = -I_k^1 + \lambda \alpha_\mathrm{i} S_k^1 k \theta, \tag{5}$$

where $\lambda = \beta/\mu$ and we have defined

$$\theta = f\alpha_\mathrm{o} \sum_{k'} \frac{(k'-1)P(k')}{\langle k \rangle} I_{k'}^1 + (1-f) \sum_{k'} \frac{(k'-1)P(k')}{\langle k \rangle} I_{k'}^0. \tag{6}$$

From here, we can write the rate equations for the rest of variables related to adopting individuals as

$$\frac{dS_k^1}{dt} = -\lambda \alpha_\mathrm{i} S_k^1 k \theta, \tag{7}$$

$$\frac{dR_k^1}{dt} = I_k^1, \tag{8}$$

while the variables affecting nonadopting individuals fulfill the rate equations

$$\frac{dI_k^0}{dt} = -I_k^0 + \lambda S_k^0 k\theta, \tag{9}$$

$$\frac{dS_k^0}{dt} = -\lambda S_k^0 k\theta \tag{10}$$

$$\frac{dR_k^0}{dt} = I_k^0. \tag{11}$$

From these equations, we immediately have

$$R_k^1(t) = \int_0^t I_k^1(t')dt', \quad R_k^0(t) = \int_0^t I_k^0(t')dt' \tag{12}$$

and

$$S_k^1(t) = e^{-\lambda k \alpha_\mathrm{i} \phi(t)}, \quad S_k^0(t) = e^{-\lambda k \phi(t)}, \tag{13}$$

where $\phi(t) = \int_0^t \theta(t')dt'$ can be written as

$$\phi(t) = \sum_{k'} \frac{(k'-1)P(k')}{\langle k \rangle} \left[f\alpha_\mathrm{o} R_{k'}^m(t) + (1-f)R_{k'}^0(t) \right] \tag{14}$$

and we assume that the initial condition consists in a very small fraction of infected individuals and thus that almost all individuals are susceptible.

In order to compute the epidemic threshold, we consider the state of the system in the infinite time limit, where $I_k^1(\infty) = I_k^0(\infty) = 0$. In this regime, the final prevalence (density of infected individuals) is given by

$$R_k^1(\infty) = 1 - S_k^1(\infty) = 1 - e^{-\lambda k \alpha_\mathrm{i} \phi_\infty} \tag{15}$$

$$R_k^0(\infty) = 1 - S_k^0(\infty) = 1 - e^{-\lambda k \phi_\infty}, \tag{16}$$

with

$$\begin{aligned} \phi_\infty &= \sum_{k'} \frac{(k'-1)P(k')}{\langle k \rangle} \left[f\alpha_\mathrm{o} R_{k'}^1(\infty) + (1-f)R_{k'}^0(\infty) \right] \\ &= \frac{\langle k \rangle - 1}{\langle k \rangle} [f\alpha_\mathrm{i} + 1 - f] - \sum_{k'} \frac{(k'-1)P(k')}{\langle k \rangle} \left[f\alpha_\mathrm{o} e^{-\lambda k \alpha_\mathrm{i} \phi_\infty} + (1-f)e^{-\lambda k \phi_\infty} \right] \equiv \Psi(\phi_\infty). \end{aligned} \tag{17}$$

A non-zero solution $\phi_\infty \neq 0$ is obtained when the condition $\left. \frac{d\Psi(\phi_\infty)}{d\phi_\infty} \right|_{\phi_\infty = 0} \geq 1$ is fulfilled, leading to

$$\lambda \frac{\langle k^2 \rangle - \langle k \rangle}{\langle k \rangle} [f\alpha_\mathrm{i}\alpha_\mathrm{o} + 1 - f] \geq 1, \tag{18}$$

which leads to the threshold value

$$\lambda_c = \frac{\langle k \rangle}{\langle k^2 \rangle - \langle k \rangle} \frac{1}{f\alpha_\mathrm{i}\alpha_\mathrm{o} + 1 - f}. \tag{19}$$

From Eqs. (15) and (16) we can recover a relation between $R_k^1(\infty)$ and $R_k^0(\infty)$, namely

$$R_k^1(\infty) = 1 - \left[1 - R_k^0(\infty)\right]^{\alpha_\mathrm{i}}, \tag{20}$$

analogous to the one obtained for homogeneous networks.

II. HETEROGENEOUS MEAN-FIELD THEORY FOR THE SIS MODEL

The HMF equations for the SIS model [6] in a general network can be written in terms of the densities of infected adopting and nonadopting individuals of degree k, I_k^1 and I_k^0, respectively, that in this case take the form

$$\dot{I}_k^1 = -I_k^1 + (1 - I_k^1)\lambda k \alpha_\mathrm{i} \theta \tag{21}$$

$$\dot{I}_k^0 = -I_k^0 + (1 - I_k^0)\lambda k \theta, \tag{22}$$

where θ, in random uncorrelated networks, is defined by

$$\theta = \sum_{k'} \frac{k'P(k')}{\langle k \rangle} \left[f\alpha_{\rm o} I^1_{k'} + (1-f) I^0_{k'} \right]. \tag{23}$$

The steady state condition, $\dot{I}^1_k = \dot{I}^0_k = 0$, corresponding to the large time behavior, leads to the equations

$$I^1_k = \frac{\lambda \alpha_{\rm i} k \theta}{1+\lambda\alpha_{\rm i} k\theta}, \qquad I^0_k = \frac{\lambda k\theta}{1+\lambda k\theta}. \tag{24}$$

Inserting these expressions into the definition of θ, we obtain the self-consistent equation

$$\theta = \sum_{k'} \frac{k'P(k')}{\langle k \rangle} \left[\frac{\lambda f \alpha_{\rm i}\alpha_{\rm o} k'\theta}{1+\lambda\alpha_{\rm i}k'\theta} + \frac{(1-f)\lambda k'\theta}{1+\lambda k'\theta} \right] \equiv \Psi(\theta). \tag{25}$$

The non-zero solution, corresponding to a finite prevalence, appears when the condition $\left.\frac{d\Psi(\theta)}{d\theta}\right|_{\theta=0} \geq 1$ is fulfilled, which leads to

$$\lambda \frac{\langle k^2 \rangle}{\langle k \rangle} \left[f\alpha_{\rm i}\alpha_{\rm o} 1 - f \right] \geq 1, \tag{26}$$

and to the threshold

$$\lambda_c = \frac{\langle k \rangle}{\langle k^2 \rangle} \frac{1}{f\alpha_{\rm i}\alpha_{\rm o} + 1 - f}. \tag{27}$$

III. SUPPLEMENTARY FIGURES

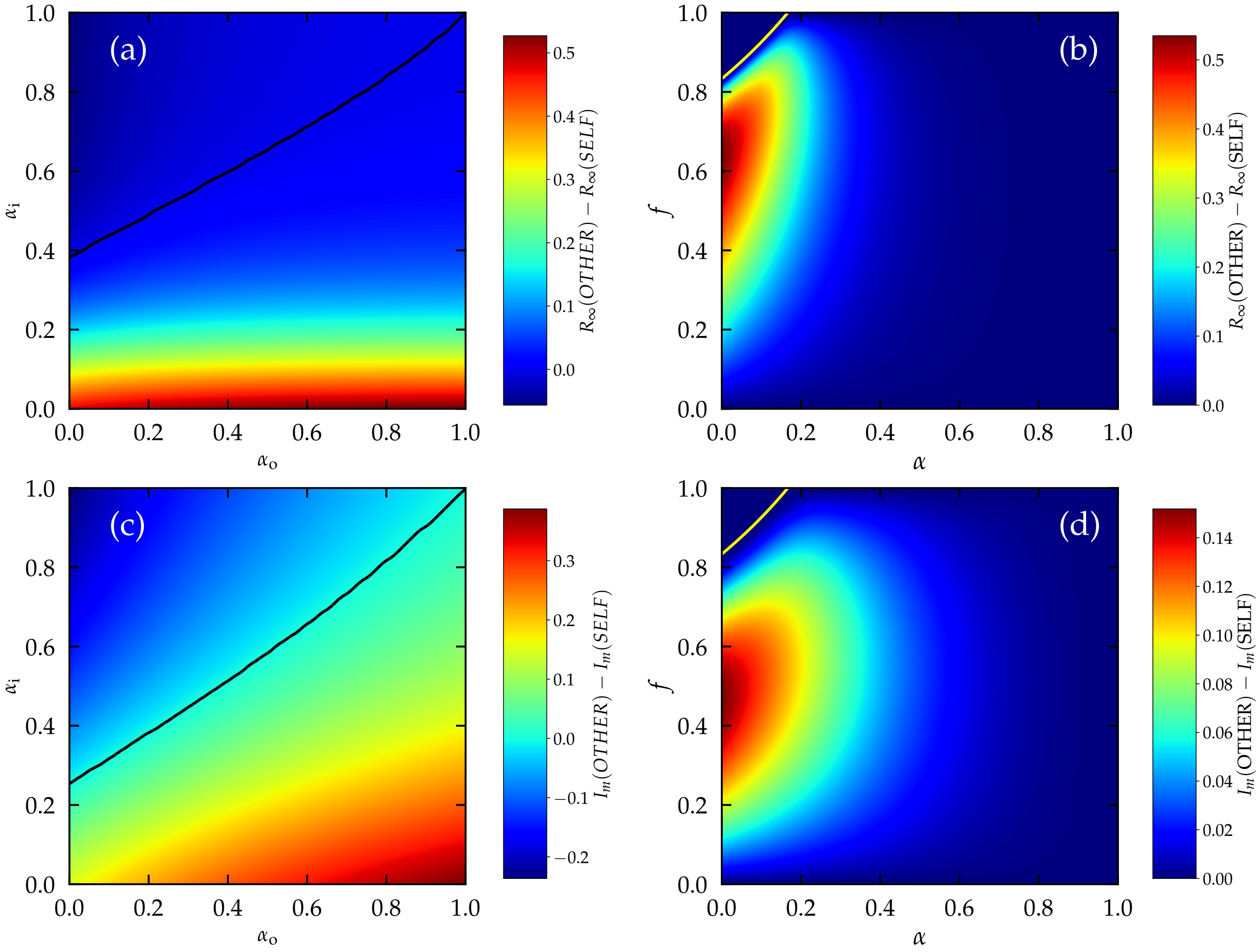

Supplementary Figure SF1. **SELF interventions are more effective than OTHER interventions at the population level.** (a) Difference between the final prevalence R_∞ for the OTHER and the SELF scenario (R_∞(OTHER) − R_∞(SELF)), as a function of α_i and α_o of the individual PI. Notice that R_∞(OTHER) depends only on α_o, and R_∞(SELF) depends only on α_i.. The solid black line indicates the zero value. (b) Difference between the final prevalence R_∞ for the OTHER and the SELF scenario as a function of the fraction f of adopters and the efficacy α of the individual PI. The solid yellow line denotes where $\lambda_c(\alpha) = \lambda$. Above it, the system is subcritical and R_∞(OTHER) − R_∞(SELF) = 0. (c) Difference between the maximum value of the incidence I_m for the OTHER and the SELF scenario (I_m(OTHER) − I_m(SELF)), as a function of α_i and α_o of the individual PI. Notice that I_m(OTHER) depends only on α_o, and I_m(SELF) depends only on α_i. The solid black line indicates the zero value. (d) Difference between the maximum value of the incidence I_m for the OTHER and the SELF scenario as a function of the fraction f of adopters and the efficacy α of the individual PI. The solid yellow line denotes where $\lambda_c(\alpha) = \lambda$. In all plots quantities greater than zero indicate that SELF interventions perform better. Solution of homogeneous MF equations for a network of degree $K = 7$ and for $\lambda = 1.0$. In panels (a) and (c) $f = 0.5$. For these parameters, in panels (a) and (c) both SELF and OTHER strategies operate above their respective thresholds.

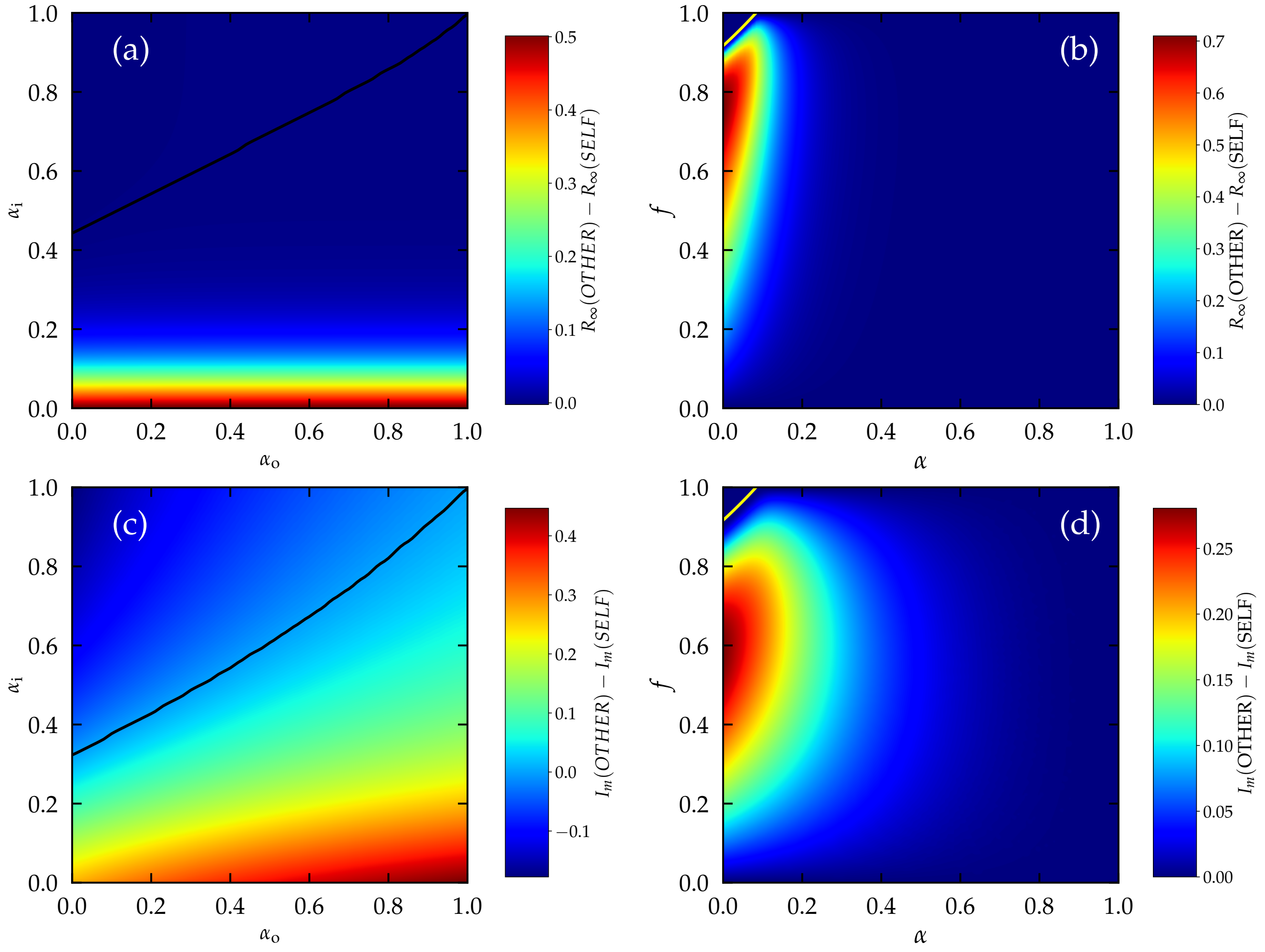

Supplementary Figure SF2. **SELF interventions are more effective than OTHER interventions at the population level.** (a) Difference between the final prevalence R_∞ for the OTHER and the SELF scenario ($R_\infty(\text{OTHER}) - R_\infty(\text{SELF})$), as a function of α_i and α_o of the individual PI. Notice that $R_\infty(\text{OTHER})$ depends only on α_o, and $R_\infty(\text{SELF})$ depends only on α_i The solid black line indicates the zero value. (b) Difference between the final prevalence R_∞ for the OTHER and the SELF scenario as a function of the fraction f of adopters and the efficacy α of the individual PI. The solid yellow line denotes where $\lambda_c(\alpha) = \lambda$. Above it, the system is subcritical and $R_\infty(\text{OTHER}) - R_\infty(\text{SELF}) = 0$. (c) Difference between the maximum value of the incidence I_m for the OTHER and the SELF scenario ($I_m(\text{OTHER}) - I_m(\text{SELF})$), as a function of α_i and α_o of the individual PI. Notice that $I_m(\text{OTHER})$ depends only on α_o, and $I_m(\text{SELF})$ depends only on α_i. The solid black line indicates the zero value. (d) Difference between the maximum value of the incidence I_m for the OTHER and the SELF scenario as a function of the fraction f of adopters and the efficacy α of the individual PI. The solid yellow line denotes where $\lambda_c(\alpha) = \lambda$. In all plots quantities greater than zero indicate that SELF interventions perform better. Solution of homogeneous MF equations for a network of degree $K = 7$ and for $\lambda = 2$. In panels (a) and (c) $f = 0.5$. For these parameters, in panels (a) and (c) both SELF and OTHER strategies operate above their respective thresholds.

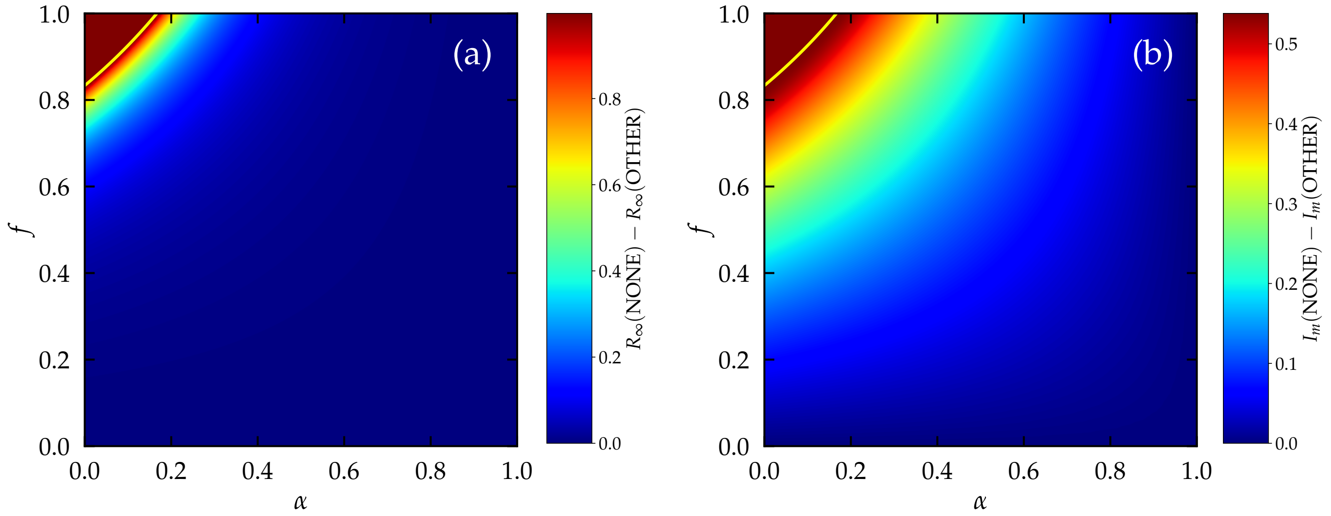

Supplementary Figure SF3. OTHER interventions flatten the curve but barely reduce prevalence with respect to no intervention. (a) Difference between the final prevalence R for the NONE and the OTHER scenario as a function of the fraction f of adopters and the efficacy α of the individual PI. (b) Difference between the maximum value of the incidence I_m for the NONE and the OTHER scenario as a function of the fraction f of adopters and the efficacy α of the individual PI. Solution of MF equations for a homogeneous network of degree $K = 7$ for $\lambda = 1$. The solid yellow lines are where $\lambda_c(\alpha) = \lambda$ for the OTHER scenario.

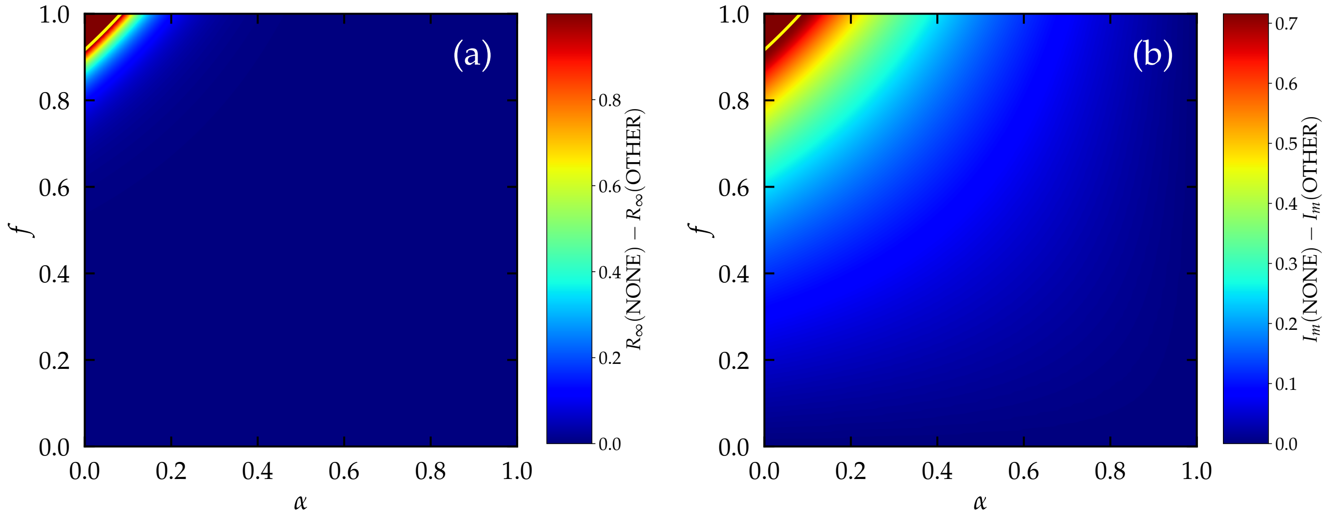

Supplementary Figure SF4. OTHER interventions flatten the curve but barely reduce prevalence with respect to no intervention. (a) Difference between the final prevalence R for the NONE and the OTHER scenario as a function of the fraction f of adopters and the efficacy α of the individual PI. (b) Difference between the maximum value of the incidence I_m for the NONE and the OTHER scenario as a function of the fraction f of adopters and the efficacy α of the individual PI. Solution of MF equations for a homogeneous network of degree $K = 7$ for $\lambda = 2$. The solid yellow lines are where $\lambda_c(\alpha) = \lambda$ for the OTHER scenario.

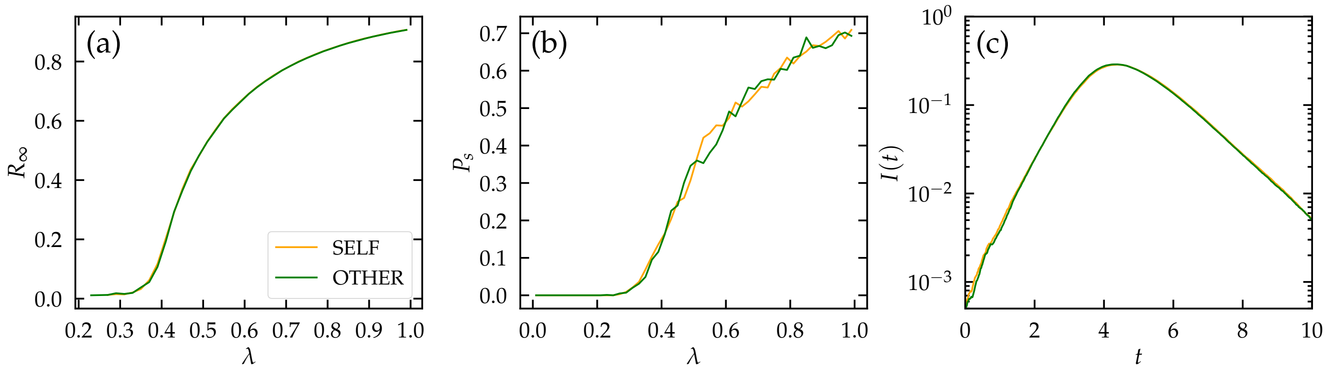

Supplementary Figure SF5. **No difference between SELF and OTHER scenarios for contact-based protecting behavior.** Simulations on a random regular graph with $K = 7$, $N = 10000$, $f = 0.5$, $\alpha = 0.2$. (a) Final prevalence R_∞ of extensive outbreaks as a function of λ, for the SELF and the OTHER scenarios. (b) Probability P_s to observe an extensive outbreak as a function of λ for the SELF and the OTHER scenarios. (c) Temporal evolution of the incidence $I(t)$ for a single realization of the dynamics; $N = 10^5$, 50 initial random infected seeds.

[1] Pastor-Satorras, R., Castellano, C., Van Mieghem, P. & Vespignani, A. Epidemic processes in complex networks. *Rev. Mod. Phys.* **87**, 925–979 (2015).
[2] Pastor-Satorras, R., Vázquez, A. & Vespignani, A. Dynamical and correlation properties of the internet. *Phys. Rev. Lett.* **87**, 258701 (2001).
[3] Dorogovtsev, S. N., Goltsev, A. V. & Mendes, J. F. F. Critical phenomena in complex networks. *Rev. Mod. Phys.* **80**, 1275–1335 (2008). URL https://link.aps.org/doi/10.1103/RevModPhys.80.1275.
[4] Moreno, Y., Pastor-Satorras, R. & Vespignani, A. Epidemic outbreaks in complex heterogeneous networks. *Eur. Phys. J. B* **26**, 521–529 (2002).
[5] Boguñá, M., Pastor-Satorras, R. & Vespignani, A. Cut-offs and finite size effects in scale-free networks. *Euro. Phys. J. B* **38**, 205–210 (2004).
[6] Pastor-Satorras, R. & Vespignani, A. Epidemic spreading in scale-free networks. *Phys. Rev. Lett.* **86**, 3200–3203 (2001).
